# Characterization and Evaluation of Aroma Quality in Doubanjiang, a Chinese Traditional Fermented Red Pepper Paste, Using Aroma Extract Dilution Analysis and a Sensory Profile

**DOI:** 10.3390/molecules24173107

**Published:** 2019-08-27

**Authors:** Zhihua Li, Ling Dong, Jin Jeon, So Young Kwon, Chi Zhao, Hyung-Hee Baek

**Affiliations:** 1 Institute of Agro-products Processing Science and Technology, Sichuan Academy of Agricultural Sciences, Chengdu 610066, China; 2 Department of Food Engineering, Dankook University, 119 Dandae-ro, Dongnam-gu, Cheonan, Chungnam 31116, Korea

**Keywords:** doubanjiang, SAFE/HS-SPME/GC-O/AEDA techniques, sotolone, β-damascenone, 3-isobutyl-2-methoxypyrazine, aroma-active compounds

## Abstract

Doubanjiang, a Chinese traditional fermented red pepper paste, is eaten worldwide for its unique flavor. The objective of this study was to evaluate the aroma quality of doubanjiang using solvent-assisted flavor evaporation (SAFE) and headspace solid-phase microextraction (HS-SPME) coupled with gas chromatography-olfactometry (GC-O) and aroma extract dilution analysis (AEDA). A total of 165 volatile compounds, belonging to 13 chemical classes, were identified. Esters and hydrocarbons were the predominant groups. Thirteen aroma-active compounds were detected by AEDA of SAFE and HS-SPME, and their odor activity values (OAVs) were calculated by dividing their concentration by their odor threshold in water. Among them, ethyl isovalerate, β-damascenone, 3-isobutyl-2-methoxypyrazine (IBMP), and sotolone had the highest OAVs (>1000). In addition, sotolone, methional, β-damascenone, 3-isobutyl-2-methoxypyrazine, ethyl isovalerate, phenylethyl alcohol and linalool had high flavor dilution (FD) factors. Sotolone, β-damascenone and 3-isobutyl-2-methoxypyrazine were identified for the first time in doubanjiang and played significant roles in its aroma quality.

## 1. Introduction

Doubanjiang is a traditional fermented red pepper paste with a Chinese protected geographical indication (PGI) that is produced with red pepper (*Capsicum annuum* L.), broad bean (*Vicia faba* L.), wheat flour, and salt [1]. It has been appreciated as a uniquely flavored seasoning for several centuries in China [2,3] and has become popular worldwide [4,5]. In general, the traditional manufacturing process consists of three phases: (1) fermentation of broad beans with salt (12–14% *w*/*w*) to make doubanjiang-meju [6], (2) fermentation of red peppers (approx. 1–2 cm) with salt (14–16% *w*/*w*) to yield red pepper moromi, and (3) aged fermentation for more than six months in the natural environment of a mix of doubanjiang-meju with red pepper moromi at a ratio of 4:6 to improve flavor and taste characteristics. The average of annual production value of doubanjiang is $1.5 billion with the export value exceeding $100 million, according to local government statistics in 2016.

The volatile composition of doubanjiang is quite complex. Several studies on the identification and quantitation of volatile compounds in doubanjiang have been reported [3,7,8,9]. However, the key aroma-active compounds in doubanjiang are still controversial; in particular, the contribution of key odorants to the overall aroma remains unclear.

Solvent-assisted flavor evaporation (SAFE) is a reliable solvent technique for volatile extraction and could direct the isolation of aroma compounds from complex food matrices [10], whereas headspace solid-phase microextraction (HS-SPME) is a solvent-free technique for volatile extraction present in the sample headspace [11]. Both solvent-assisted and solvent-free extraction methods have been widely applied to the analysis of aroma compounds in many kinds of foods, such as doenjang [12], daqu [13], various sauce [14] and gochujang [15]. Additionally, gas chromatography-olfactometry (GC-O), aroma extract dilution analysis (AEDA), and omission experiments have been used to investigate the key odorants extensively and successfully in many foods such as soy sauce [11,14], gochujang [15], and light aroma type Chinese liquor [16].

The aims of this study were to (1) profile the volatile compounds in doubanjiang using SAFE and HS-SMPE, coupled with gas chromatography-mass spectrometry (GC-MS), (2) identify the aroma-active compounds using AEDA and GC-O, and (3) evaluate the most odor-active compounds in doubanjiang. The results will provide a better understanding of the key aroma-active compounds in doubanjiang and could be beneficial in improving the flavor of doubanjiang in the industry.

## 2. Results and Discussion

### 2.1. Volatile Compounds Identified in Doubanjiang 

Volatile compounds in doubanjiang were extracted using SAFE and HS-SPME and identified using GC-MS. A total of 168 volatile compounds belonging to 13 chemical classes were identified. As shown in Table 1, hydrocarbons and esters were the predominant groups, each containing 30 and 26 volatile compounds, respectively, followed by alcohols, ketones, and acids, with each group including 27, 19, and 13 volatile compounds, respectively. For the SF-A sample, the most abundant volatile group (>5% average relative areas) was alcohols (17.86%), followed by phenols (10.08%) and esters (10.02%), while for the XH-B and XH-C commercial samples, esters (9.41% and 15.04%, respectively) and alcohols (6.14% and 7.01%, respectively) were major components (Table 2). This difference might be due to the different manufacturing conditions, as the SF-A sample was produced by a different company than XH-B and XH-C.

### 2.2. Aroma-Active Compounds in Doubanjiang

Studies of the aroma-active compounds of doubanjiang are limited, despite its Chinese Protected Geographical Indication (PGI) status and as traditional fermented food. AEDA was performed for SAFE and HS-SPME. Table 3 shows the aroma-active compounds identified in doubanjiang by SAFE and HS-SPME. A total of 13 aroma-active compounds were detected, including eight by SAFE and eight by HS-SPME. Of these, five aroma-active compounds were verified by both SAFE and HS-SPME. Among them, the seasoning-like compound 3-hydroxy-4,5-dimethyl-2(5H)-furanone (sotolone) and cooked potato-like compound 3-(methylthio) propanal (methional) were the most intense aroma-active compounds (log_3_FD = 6), followed by 3-isobutyl-2-methoxypyrazine (bell pepper), and β-damascenone (fruit) (log_3_FD = 5), and phenylethyl alcohol (rose) (log_3_FD = 4) identified by SAFE. Ethyl 3-methylbutanoate (ethyl isovalerate) has the highest log_2_FD value (log_2_FD = 4), followed by limonene (fragrant), linalool (bergamot), β-damascenone (fruit), 3-isobutyl-2-methoxypyrazine(bell pepper), and 2-methoxyphenol (bacon) (log_2_FD = 4) identified by HS-SPME. This difference might be due to the extraction method [15]. Compared to SAFE, the selectivity of the fiber also leads to the profiling of different volatile compounds [17].

Sotolone has already been confirmed in previous studies as a key odor-active compound and significantly imparts caramel-like and seasoning-like notes in soy sauce [17,18,19]. Sotolone is a furan derivative compound generated by nonenzymatic pathways, which include an aldol condensation step to form the branched six-membered carbon skeleton [20]. Although two furanones, 4-hydroxy-2,5-dimethyl-3(2H)-furanone (HDMF) and 4-hydroxy-2(or 5)-ethyl-5(or 2)-methyl-3(2H)-furanone (HEMF) have been reported as the key aroma compounds in doubanjiang (also aka horse bean-chili-paste) [3], sotolone was the most intense aroma-active compound and identified for the first time in doubanjiang.

3-(Methylthio) propanal (methional), which imparts cooked potato-like aroma note, is identified as an important aroma-active compound for most paste and sauce products such as soy sauce [14], doubanjiang [3] and gochujang, a Korean fermented red pepper paste [15]. Methional could be formed by Strecker degradation of methionine [21].

β-Damascenone, exhibiting fruity and sweet notes, is an important odorant in modern perfumery and a major contribution to wine aromas [22]. β-Damascenone has a very low odor threshold in water (2 ng/L) (Table 4) and may be formed by acid-catalyzed hydrolysis of certain plant-derived metabolites under heat treatments and low pH during the manufacturing process [23]. Moreover, synergistic phenomena have also been identified, for instance, reducing the orthonasal detection thresholds of linalool and raising the detection threshold of 3-isobutyl-2-methoxypyrazine in the presence of β-damascenone [22]. To our knowledge, this is the first time that β-Damascenone is identified in doubanjiang.

3-Isobutyl-2-methoxypyrazine has been reported in bell and chili peppers, grapes and wines, and fermented red pepper food such as gochujang, a Korean fermented red pepper paste [15]. The odor threshold of IBMP has an extremely low value of 2 ng/L in water, contributing to a characteristic bell pepper, green pepper and gochujang-like aroma [15]. l-Leucine was speculated to be a precursor in regulating IBMP biosynthesis in grape berry [24]. 3-Isobutyl-2-methoxypyrazine was also identified for the first time in doubanjiang to our knowledge.

Sotolone, β-damascenone and 3-isobutyl-2-methoxypyrazine were identified for the first time in doubanjiang and validated by the mass spectrum, RI (DB-wax and/or DB-5ms columns), aroma properties and authentic compounds.

### 2.3. Quantitation of the Key Odorants and Calculation of Odor Activity Values (OAVs)

Table 4 lists the calculated mean values for each volatile compound in doubanjiang. Among these odorants, 3-methylbutanoic acid had the highest concentration in doubanjiang (144.8 μg/kg), followed by phenylethyl alcohol (129.2 μg/kg), acetic acid (103.2 μg/kg), ethyl isovalerate (62.3 μg/kg), limonene (32.4 μg/kg), linalool (33.2 μg/kg), methional (32 μg/kg) and 2-methoxyphenol (27.2 μg/kg). Odor activity values (OAVs) were used to estimate the contribution of individual aroma compounds to the overall flavor of doubanjiang. As shown in Table 4, the concentrations of the selected key aroma volatiles exceeded their estimated odor threshold, suggesting that they may be responsible for the doubanjiang aroma. Ethyl 3-methylbutanoate showed the highest OAV, exceeding its threshold (0.01 μg/L in water) by a factor of 6229, followed by β-damascenone, 3-isobutyl-2-methoxypyrazine and sotolone, with OAVs of 3345, 2490 and 1602, respectively. These results correlated well with the conclusion that ethyl 3-methylbutanoate had a high OAV in doubanjiang [3]. β-Damascenone was the key odorant in red wine and Chinese liquor, with a high OAV, and significantly imparted the overall aroma of Chinese liquor [16]; sotolone showed the highest OAVs in soy sauce [19].

### 2.4. The effects of Selected Key Odorants on the Sensory Profiles of Doubanjiang

Sensory evaluation tests were used to access the effect of selected key odorants on the overall aroma of doubanjiang. The effects of spiking certain volatiles alone on flavor descriptors are shown in Table 5. Significant difference was observed compared with the unspiked control, which further confirmed that these key active-aromas played an important role in the flavor of doubanjiang. Doubanjiang has a quite complex food matrix, from which flavor release not only depends on the nature of the aroma compounds but also the interaction between sensory attributes of aroma compounds or with the nonvolatile constituents of the food matrix. For example, spiking acetic acid, 3-methylbutanoic acid and methional separately from doubanjiang did not impart the overall aroma, although methional has a relatively high OAV (160), whereas adding sotolone to doubanjiang not only imparted a caramel-like aroma but also increased the fruity aroma. 

## 3. Materials and Methods

### 3.1. Doubanjiang Samples

Three commercial doubanjiang samples (labeled as SF-A, XH-B, and XH-C), manufactured by two doubanjiang companies located in Pixian city (Sichuan province, China-Latitude 30.79, Longitude 103.89), were used in this study. SF-A was produced in 2015 (aged 3 years), while XH-B and XH-C were produced by the same company in 2014 (aged 2 and 5 years, respectively). All three samples were collected from the company directly. The label on these products indicated that they contained ca. 47.8% red pepper, 26.1% broad beans, 5% wheat flour, and 16.7% salt.

### 3.2. Chemicals

Authentic standards, including acetic acid, phenylacetaldehyde, β-damascenone, dimethyl disulfide, decanal, 4-ethyl-2-methoxyphenol, ethyl benzoate, ethyl lactate, ethyl 3-methylbutanoate, ethyl phenylacetate, furfuryl alcohol, 2-methoxyphenol, limonene, linalool, methional, methyl salicylate, 3-methylbutanal, nonanal, phenylethyl alcohol, sotolone, 3-isobutyl-2-methoxypyrazine (IBMP), 3-methylbutanoic acid and isoamyl acetate were purchased from Sigma-Aldrich Chemical Co. (St. Louis, MO, USA) or Sigma-Aldrich China Co. (Shanghai, China).

### 3.3. Extraction of Volatile Components from Doubanjiang

#### 3.3.1. Solvent-Assisted Flavor Evaporation (SAFE)

Five gram of each doubanjiang sample was mixed with 100 mL double distilled water (ddH2O), shaken at 250 rpm for 1.5 h, and filtered through a filter paper (Whatman, United Kingdom) under vacuum using a Büchner funnel. Volatiles from the total filtration containing internal standard (3-heptanol, 48.585 μg) were isolated using a solvent-assisted flavor evaporation (SAFE) unit (ACE Glass Inc., Vineland, NJ, USA) at 25 °C for 1.5 h under vacuum (10 ^−6^ torr), according to the Seo et al. [25] method with some modifications. After distillation, the extract was obtained by sequential extraction with dichloromethane solvent (15 mL, 15 mL, and 20 mL) at 250 rpm for 1 h. After extraction, removal of the water was carried out by freezing at −20 °C overnight and followed by dehydrating over anhydrous sodium sulfate. A gentle N_2_ stream method was used to concentrate the extract to 200 μL. Extractions were performed in duplicate.

#### 3.3.2. Headspace Solid-Phase Microextraction (HS-SPME)

The volatile constituents of doubanjiang were extracted using two fibers, namely 75 μm carboxen/PDMS (CAR/PDMS) and 50/30 μm DVB/CAR/PDMS (Supelco Co., Bellefonte, PA, USA) with ca. 80% and 60% global perception similarity to doubanjiang, respectively, according to the direct GC-O method [15,17]. Briefly, 5.0 g of doubanjiang sample mixed with 10 mL of deodorized distilled water (DDW) was placed into a 20-mL vial, and balanced at 40 °C for 30 min in a water bath under stirring. The SPME fiber was then inserted into the vial and exposed to the headspace to extract volatiles at 40 °C for 30 min. After extraction, the fiber was inserted into the injection port of gas chromatograph (GC) for thermal desorption with the same parameters described by our previous method [26]. Extractions were performed in triplicate.

### 3.4. GC-MS and GC-O Analysis

#### 3.4.1. GC-Mass Spectrometry (GC-MS) Conditions

An Agilent 6890N gas chromatograph coupled with a mass spectrometer (MS 5973N, Agilent Technologies, Santa Clara, CA) was used in this study. First, one μL of SAFE extracts or SPME fiber extracts was injected in splitless mode onto both DB-wax column (60 m length, 0.25 mm i.d., 0.25 μm film thickness, J&W Scientific, Folsom, CA, USA) and DB-5ms column (60 length, 0.25 mm i.d., 0.25 μm film thickness, J&W Scientific, Folsom, CA, USA), respectively. Helium was used as the carrier gas with flow rate of 1 mL/min. The temperature program parameters of mass spectrometer were the same as our newly published paper [26].

#### 3.4.2. Gas Chromatography-Olfactometry (GC-O)

The GC-olfactometry (GC-O) system was composed of a Varian 3800 GC (Varian Instrument Group, Walnut Creek, CA) coupled to a sniffing port (ODO II, SGE International, Ringwood, Australia) and a flame ionization detector (FID). Analytical conditions were performed following our published paper [27]. In brief, the conditions of volatile compounds extracted by both SAFE and HS-SPME were the same as GC-MS. Two different columns, namely, DB-wax column (30 m length, 0.25 mm i.d., 0.25 μm film thickness, J&W Scientific, Folsom, CA, USA) and DB-5ms column (30 length, 0.25 mm i.d., 0.25 μm film thickness, J&W Scientific, Folsom, CA, USA),respectively, were used for GC-O. Each SPME fiber was injected into the GC injector for 10 min. The GC conditions were the same as GC-MS: oven temperature program was from 40 °C to 200 °C at a rate of 5 °C/min with initial 5 min and final 10 min, respectively. The flow rate of the carrier gas helium was 1.4 mL/min. Injector and detector temperatures were 280 °C and 250 °C, respectively. GC-O was performed in triplicate.

To determine the most aroma-active components, aroma extract dilution analysis (AEDA) was carried out by serially diluting (1:3) the SAFE extracts using the same diluent. The flavor dilution (FD) factor is determined as the highest dilution where a volatile odor can still be perceived [27]. The HS-SPME extracts were stepwise diluted (10 mL DDW mixed with 5 g, 2.5 g, 1.25 g and 0.625 g of doubanjaing, respectively, were put into a 20-mL vial) and then balanced at 40 °C for 30 min. The sample dilution (SD) factor is determined as the highest sample dilution where an odorous substance can be perceived [28].

### 3.5. Compound Identification, Quantitation of the Key Odorants and Calculation of Odor Activity Values (OAVs)

Compounds were identified based on a comparison of the WILEY 7.0 mass spectral library, retention indices (RIs) and authentic standard compounds [26]. The retention indice (RI) values were calculated on DB-wax and DB-5ms columns using C_10_–C_22_ and C_8_–C_20_ as the external reference, respectively.

The selective ion monitoring (SIM) mode was used to quantitate the concentration of a compound in the SAFE concentrate mentioned above with 8-point external standard curves. The identified key aroma compounds were confirmed by authentic chemicals, and the standard curve for individual aroma compounds was obtained by calculating the response factor of known concentrations on GC-MS. The unit of concentration in the SAFE concentrate (μg/L) was converted to micrograms per kilogram (μg/kg, doubanjiang) according to the weight of sample used. For each compound, the OAVs were calculated by dividing the compound concentration in the samples by the mean values of its estimated odor threshold. Relative quantification measurement was also performed for all the volatile compounds, using 3-heptanol as the internal standard.

### 3.6. Sensory Evaluation 

To access the effects of selected key aroma-active compounds on the overall aroma of doubanjiang, sensory evaluation was performed according to a previous method [11] by eight panelists (4 females, 4 males, aged 22–38) recruited from the Institute of Agro-products Processing Science and Technology, Sichuan Academy of Agricultural Sciences. Briefly, a total of 8 attributes containing ethanol (alcohols), acetic acid (sour), methional (cooked potato), 4-ethyl-2-methoxyphenol (burnt), phenylacetaldehyde (sweet), soy sauce (caramel-like), and ethyl acetate (fruity) [11,29] were used to characterize the sensory properties of the doubanjiang samples. The doubanjiang was spiked with 2–3 times concentration of certain key aroma-active compounds, and the panelists were asked to evaluate the odor intensity compared to unspiked samples at room temperature (25 ± 2 °C) using a line scale of 0–9, in which 0 was the lowest intensity without perception and 9 was the highest intensity. All the panelists had experience in the theory and practical application of doubanjiang, and could differentiate differences in doubanjiang’s flavor.

### 3.7. Statistical Analysis

Univariate statistical analysis (one-way ANOVA) was used to determine significance between spiked and unspiked individual key aroma-active compounds using the statistical software OriginPro 8 (OriginLab Corporation, Northampton, MA, USA).

## 4. Conclusions

In summary, a total of 165 volatile compounds were identified using SAFE and/or HS-SPME. Thirteen most important odorants were detected, including eight by SAFE-GC-O and eight by HS-SPME-GC-O; of these, five aroma-active compounds were identified by both methods. Sotolone, β-damascenone and 3-isobutyl-2-methoxypyrazine, contributing to caramel-like, fruity, and red pepper aromas, respectively, were identified for the first time in doubanjiang. These compounds played key roles in the aroma quality of doubanjiang. In addition, methional, ethyl 3-methylbutanoate, phenylethyl alcohol, 2-methoxyphenol, 3-methylbutanal, and phenylacetaldehyde also played important roles in the aroma quality of doubanjiang. These results will be useful to improve the aroma quality of this traditional food.

## Figures and Tables

**Table 1:** Volatile flavor compounds identified in doubanjiang.

No.	RI ^a^		Compounds ^b^	Odor Description ^c^	Identification ^d^
	DB-5ms	DB-wax			
***Esters (26)*^e^**					
1	<800	<1000	ethyl acetate ^3^	balsamic, pineapple, sweet	MS, RI
2	816	1351	ethyl lactate ^1^	butter, floral, sweet	MS, RI, Std, O
3	850	1075	ethyl 3-methylbutanoate ^3^	apple, pineapple, sweet	MS, RI, Std, O
4	872	1134	isoamyl acetate ^2^	apple, banana, sweet	MS, RI, Std, O
5	1099	1520	ethyl sorbate ^3^	apple, honey	MS, RI
6	1176	1688	ethyl benzoate ^3^	camomile, flower, fruit	MS, RI, Std, O
7	1201	1804	methyl salicylate ^1^	caramel, peppermint	MS, RI, Std, O
8	1240	-	ethyl phenylacetate ^2^	floral, rose, sweet	MS, RI
9	1242	-	linalyl acetate ^2^	fruit, resinous, sweet	MS, RI
10	1247	-	ethyl phenylacetate ^1^	floral, rose, sweet	MS, RI, Std, O
11	1252	1837	phenethyl acetate ^3^	floral, rose, tobacco	MS, RI
12	1349	-	neryl acetate ^2^	floral, fruit, sweet	MS, RI
13	1368	-	geranyl acetate ^2^	lavender, rose, sweet	MS, RI
14	1522	-	methyl laurate ^1^	coconut, fat	MS, RI
15	1591	1849	ethyl laurate ^1^	floral, fruit, leaf, nut	MS, RI
16	1651	-	methyl hydrojasmonate ^1^	-	MS, RI
17	1720	2016	methyl myristate ^1^	fat, honey, oil, orris	MS, RI
18	1789	-	ethyl myristate ^1^	ether, pleasant, soap	MS, RI
19	1818	2038	isopropyl myristate ^1^	cherry, cinnamon, waxy	MS, RI
20	1850	2153	ethyl pentadecanoate ^1^	-	MS, RI
21	1989	>2200	ethyl palmitate ^1^	Wax	MS, RI
22		<1000	isopropenyl acetate ^2^	-	MS, RI
23	-	>2200	ethyl 9-hexadecenoate ^1^	-	MS, RI
24	-	>2200	ethyl oleate ^1^	-	MS, RI
25	-	>2200	methyl palmitate ^1^	-	MS, RI
26	-	>2200	methyl linoleate ^1^	-	MS, RI
***Hydrocarbons (30)***					
27	<800	1045	toluene ^1^	glue, paint, solvent	MS, RI
28	1029	1193	limonene ^3^	balsamic, fragrant, fruit	MS, RI, Std, O
29	1085	-	terpinolene ^2^	pine, plastic, sweet	MS, RI
30	1199	1772	naphthalene ^1^	mothball, tar	MS, RI
31	1237	-	4-methylcyclopentene ^2^	-	MS, RI
32	1363	-	2-methyltridecane ^3^	-	MS, RI
33	1375	-	ylangene ^2^	fruit	MS, RI
34	1381	-	copaene ^2^	spice, wood	MS, RI
35	1386	-	longicyclene ^2^	-	MS, RI
36	1391	-	β-elemen ^2^	-	MS, RI
37	1434	-	zingiberene ^2^	fresh, mint, sharp, spice	MS, RI
38	1447	-	thujopsene ^2^	-	MS, RI
39	1463	-	2-methyltetradecane ^1^	-	MS, RI
40	1471	-	α-himachalene ^3^	-	MS, RI
41	1475	-	cyclododecane ^1^	-	MS, RI
42	1479	-	α-selinene ^2^	wood	MS, RI
43	1498	-	eremophilene ^2^	wood	MS, RI
44	1501	1723	α-muurolene ^1^	wood	MS, RI
45	1509	1736	valencene ^1^	citrus, green, oil, wood	MS, RI
46	1519	-	γ-cadinene ^2^	wood	MS, RI
47	1526	-	calamenene ^2^	clove, floral, herb, spice	MS, RI
48	1548	-	α-calacorene ^2^	wood	MS, RI
49	1564	-	2-methylpentadecane ^3^	-	MS, RI
50	1612	>2200	fluorene ^1^	-	MS, RI
51	1660	-	2-methylhexadecane ^1^	-	MS, RI
52	1677	-	cadalene ^2^	-	MS, RI
53	1700	-	heptadecane ^1^	alkane, biting, pungent	MS, RI
54	1731	-	guaiazulene ^1^	-	MS, RI
55	-	1268	styrene ^1^	balsamic, rubber, solvent	MS, RI
56	-	1743	β-selinene ^3^	herb	MS, RI
***Alcohols (27)***					
57	<800	1583	2,3-butanediol ^3^	-	MS, RI
58	<800	1209	3-methyl-1-butanol ^3^	banana, cocoa, floral	MS, RI
59	872	1356	hexanol ^3^	Green, grassy, fatty, leafy	MS, RI
60	907	1410	2-butoxyethanol ^1^	-	MS, RI
61	971	1458	1-heptanol ^1^	nutty	MS, RI
62	1029	1492	2-ethyl-1-hexanol ^3^	citrus, green, oil, rose	MS, RI
63	1042	1896	benzyl alcohol ^3^	floral, roasted bread	MS, RI
64	1101	1551	linalool ^3^	bergamot, lavender, rose	MS, RI, Std, O
65	1120	1933	phenylethyl alcohol ^3^	fruit, rose, sweet apple	MS, RI, Std, O
66	1183	1616	terpinen-4-ol ^3^	earth, must, wood	MS, RI
67	1204	1709	α-terpineol ^1^	fresh, mint, oil, sweet	MS, RI
68	1249	-	citronellol ^1^	citrus, green, rose	MS, RI
69	1260	2004	2-phenylbutane-1-ol ^1^	-	MS, RI
70	1565	2043	nerolidol ^1^	fir, linoleum, pine	MS, RI
71	1624	-	cedrol ^2^	sweet	MS, RI
72	-	1097	2-methyl-1-propanol ^2^	apple, cocoa, fusel, malt	MS, RI
73	-	1253	pentanol ^1^	unpleasant	MS, RI
74	-	1316	4-methyl-1-pentanol ^3^	-	MS, RI
75	-	1389	3-hexen-1-ol ^3^	bell pepper, grass, herb	MS, RI
76	-	1391	cis-3-hexen-1-ol ^2^	bell pepper, grass, herb	MS, RI
77	-	1395	3-octanol ^3^	citrus, mushroom, oil	MS, RI
78	-	1466	6-methyl-5-hepten-2-ol ^1^	-	MS, RI
79	-	1561	octanol ^1^	Orange peel	MS, RI
80	-	1621	cyclooctyl alcohol ^1^	-	MS, RI
81	-	1650	levomenthol ^1^	-	MS, RI
82	-	2027	2-hepten-4-ol ^1^	-	MS, RI
83	-	2163	2-phenoxy-ethanol ^1^	pleasant	MS, RI
***Ketones (19)***					
84	<800	-	acetoin ^3^	green pepper, rancid	MS, RI
85	937	1457	2-cyclohexen-1-one ^1^	solvent, unpleasant	MS, RI
86	1151	1716	4-oxoisophorone ^1^	citrus, honey, must	MS, RI
87	1226	-	eucarvone ^2^	-	MS, RI
88	1246	-	D-carvone ^2^	minty	MS, RI
89	1391	1803	β-damascenone ^3^	fruit, grape, sweet, floral	MS, RI, Std, O
90	1431	-	α-ionone ^1^	floral, violet, wood	MS, RI
91	1441	1868	geranyl acetone ^3^	green, hay, magnolia	MS, RI
92	1488	1961	β-ionone ^1^	floral, raspberry, seaweed	MS, RI
93	1551	>2200	dihydroactinidiolide ^1^	-	MS, RI
94	1715	-	blumenol C ^1^	-	MS, RI
95	1837	-	hexahydrofarnesyl acetone ^3^	-	MS, RI
96	-	1262	3-octanone ^1^	butter, herb, mold, resin	MS, RI
97	-	1306	cyclohexanone ^1^	bitter, peppermint	MS, RI
98	-	1314	1-hydroxy-2-propanone ^1^	mushroom	MS, RI
99	-	1347	6-methyl-5-heptene-2-one ^1^	mushroom, pepper, rubber	MS, RI
100	-	1418	thujone ^2^	-	MS, RI
101	-	1610	6-methylhepta-3,5-dien-2-one ^1^	-	MS, RI
102	-	1819	2-tridecanone ^1^	harsh, herb, oil, spice	MS, RI
***Acids (13)***					
103	-	1459	acetic acid ^2^	pungent, sour, vinegar	MS, RI, Std, O
104	-	1576	2-methylpropanoic acid ^2^	burnt, cheese, rancid	MS, RI
105	-	1637	butanoic acid ^2^	butter, cheese, rancid	MS, RI
106	-	1678	3-methylbutanoic acid ^2^	cheese, rancid, sweat	MS, RI, Std, O
107	-	1811	4-methylpentanoic acid ^1^	floral, sour, sweat	MS, RI
108	-	1854	hexanoic acid ^1^	acid, pungent, rancid	MS, RI
109	1113	1956	2-ethylhexanoic acid ^1^	soapy	MS, RI
110	-	1960	heptanoic acid ^1^	rancid, sour, sweat	MS, RI
111	-	2064	octanoic acid ^1^	acid, fat, rancid, sweat	MS, RI
112	-	2170	nonanoic acid ^1^	fat, green, sour	MS, RI
113	-	>2200	benzoic acid ^2^	pungent, sour, urine	MS, RI
114	-	>2200	decanoic acid ^1^	dust, fat, rancid, sweat	MS, RI
115	-	>2200	lauric acid ^1^	fat, fruit, metal, wax	MS, RI
***Nitrogen-containing compounds (10)***					
116	886	1258	2,6-dimethylpyridine ^1^	green, coffee, nutty	MS, RI
117	915	1345	2,6-dimethylpyrazine ^2^	cocoa, coffee, roasted nut	MS, RI
118	1068	1996	2-acetylpyrrole ^1^	herbal, nutty, anisic,	MS, RI
119	1087	-	tetramethylpyrazine ^1^	cocoa, coffee, roast	MS, RI
120	-	1045	1-hexanamine ^2^	-	MS, RI
121	-	1277	methylpyrazine ^1^	cocoa, popcorn, roasted	MS, RI
122	-	1396	2-ethyl-5-methylpyrazine ^1^	fruit, roast, sweet	MS, RI
123	-	1421	N,N-dimethylacetamide ^1^	amine, burnt, oil	MS, RI
124	-	1510	3-isobutyl-2-methoxypyrazine ^3^	bell pepper, green pepper	MS, RI, Std, O
125	-	1700	N-methylpyrrolidinone ^1^	-	MS, RI
***Phenols (8)***					
126	1091	1883	2-methoxyphenol ^3^	bacon, medicine, phenol	MS, RI, Std, O
127	1164	2193	4-ethylphenol ^3^	medicine, phenol, stable	MS, RI
128	1197	-	estragole ^2^	anise, licorice	MS, RI
129	1280	2050	4-ethyl-2-methoxyphenol ^3^	medicine, phenol, smoke	MS, RI, Std, O
130	1294	1849	anethole ^3^	anise	MS, RI
131	-	1979	4-methylguaiacol ^1^	clove, phenol, smoke	MS, RI
132	-	2023	phenol ^2^	medicine, phenol, spice	MS, RI
133	-	2188	3-allyl-6-methoxyphenol ^1^	-	MS, RI
***Aldehydes (8)***					
134	<800	<1000	3-methylbutanal ^2^	cocoa, malt, pungent	MS, RI, Std, O
135	1364	-	4-methyl-2-phenyl-2-pentenal ^2^	cocoa	MS, RI
136	1050	1668	phenylacetaldehyde ^3^	berry, honey, nut, pungent	MS, RI, Std, O
137	1106	1403	nonanal ^1^	citrus, paint, pungent	MS, RI, Std, O
138	1493	2098	5-methyl-2-phenyl-2-hexenal ^3^	cocoa	MS, RI
139	-	1089	hexanal ^1^	green, beany	MS, RI
140	-	1508	decanal ^1^	floral, fried, orange peel	MS, RI, Std, O
141	-	1547	benzaldehyde ^3^	burnt sugar, roasted pepper	MS, RI
***Lactones (7)***					
142	915	1660	γ-butyrolactone ^3^	caramel, fruit, roasted nut	MS, RI
143	956	1638	γ-valerolactone ^1^	herb, sweet	MS, RI
144	963	-	tetrahydro-2-pyranone ^1^	sweet	MS, RI
145	1046	-	pantolactone ^1^	burnt, roasted bread	MS, RI
146	1367	2058	γ-nonalactone ^1^	apricot, cocoa, coconut	MS, RI
147	1541	-	dihydroactinolide ^2^	-	MS, RI
148	-	1698	lavender lactone ^1^	-	MS, RI
***Furans (6)***					
149	858	1672	furfuryl alcohol ^3^	burnt, caramel, cooked	MS, RI, Std, O
150	965	1593	5-methylfurfural ^1^	caramel, roasted garlic, spice	MS, RI
151	1070	-	cis-linalool oxide ^2^	earth, flower, nut, root	MS, RI
152	1169	2186	sotolone ^3^	savory, spice, soy sauce	MS, RI, Std, O
153	-	1484	furfural ^2^	baked potatoes, bread	MS, RI
154	-	1523	2-acetylfuran ^1^	balsamic, cocoa, coffee	MS, RI
***Sulfur-containing compounds (5)***					
155	911	1473	methional ^1^	cooked potato, soy	MS, RI, Std, O
156	986	1732	methionol ^1^	cooked potato, earth, soy	MS, RI
157	1529	-	2,5-bis(2-methylpropyl)thiophene ^1^	-	MS, RI
158	-	1083	dimethyl disulfide ^2^	garlic, onion, putrid	MS, RI, Std, O
159	-	1403	dimethyl trisulfide ^2^	onion, sulfur, sweat	MS, RI
***Ethers (5)***					
160	1004	1631	2-(2-ethoxyethoxy)ethanol ^1^	-	MS, RI
161	1320	-	Theaspirane (A or B) ^2^	honey	MS, RI
162	-	1207	1,8-cineole ^2^	eucalyptol, mint, sweet	MS, RI
163	-	1581	methyl carbitol ^1^	-	MS, RI
164	-	1167	cyclohexene epoxide ^1^	-	MS, RI
***Miscellaneous compounds (1)***					
165	1571	-	p-chloroaniline ^1^	-	MS, RI

^a^ Retention indices (RI) were determined on DB-5ms and DB-wax column with C_8_–C_20_ and C_10_–C_22_ as external reference, respectively. “-” Not determined. ^b^ Superscript numbers “1” and “2” indicates that the corresponding volatile compound is identified by SAFE and HS-SPME, respectively, “3” means that the corresponding volatile compound is identified by both methods. ^c^ Odor descriptions are based on VCF database (http://www.vcf-online.nl/VcfHome.cfm). “-” means not reported. ^d^ MS, compounds identified by MS spectra; RI, compounds identified by comparison with the retention indices (RIs) on DB-5ms and/or DB-wax column in the NIST Chemistry WebBook (http://webbook.nist.gov/); Std, confirmed by authentic compound. O, compounds identified by the odor confirmation. ^e^ Numbers in each group given in parentheses indicate the number of identified compounds.

**Table 2:** Relative quantification analysis of chemical groups after SAFE in three doubanjiang samples.

Group	SF-A	XH-B	XH-C
esters	10.02 ± 2.87 ^a^	9.41 ± 2.73	15.04 ± 2.56
hydrocarbons	4.04 ± 0.36	1.77 ± 0.42	2.56 ± 0.12
alcohols	17.86 ± 2.54	6.14 ± 1.24	7.01 ± 0.51
ketones	4.32 ± 0.11	1.97 ± 0.25	2.63 ± 0.50
Acids	4.43 ± 0.95	2.35 ± 1.16	3.14 ± 1.29
nitrogen-containing compounds	1.07 ± 0.08	0.82 ± 0.14	0.83 ± 0.03
phenols	10.08 ± 0.53	3.79 ± 0.34	3.75 ± 0.33
aldehydes	2.87 ± 0.41	1.16 ± 0.28	1.56 ± 0.26
lactones	2.35 ± 0.27	1.21 ± 0.12	1.28 ± 0.15
furans	1.13 ± 0.08	0.55 ± 0.18	0.66 ± 0.13
sulfur-containing compounds	0.71 ± 0.02	0.58 ± 0.16	0.87 ± 0.08
ethers	0.71 ± 0.04	0.26 ± 0.03	0.11 ± 0.02
Miscellaneous compounds	0.33 ± 0.05	0.15 ± 0.12	0.30 ± 0.05

^a^ The relative areas of each chemical group compared their total peak areas to that of 3-heptanol (48.585 μg), an internal standard compound, were used as relative quantification analysis. Mean ± standard deviation.

**Table 3:** Aroma-active compounds detected in doubanjiang by SAFE and HS-SPME-AEDA.

No.	RI		Compounds	Log_3_FD ^a^ (SAFE)	Log_2_SD ^b^ (SPME)
	DB-5ms	DB-wax			
3	850	1075	ethyl 3-methylbutanoate	3	4
28	1029	1193	limonene	-	3
64	1101	1551	linalool	3	3
65	1120	1933	phenylethyl alcohol	4	-
89	1391	1803	β-damascenone	5	3
103	-	1459	acetic acid	-	2
106	-	1678	3-methylbutanoic acid	1	-
124	-	1510	3-isobutyl-2-methoxypyrazine	5	3
126	1091	1883	2-methoxyphenol	-	3
134	<800	<1000	3-methylbutanal	-	1
136	1050	1668	phenylacetaldehyde	2	-
152	1169	2186	sotolone	6	-
155	911	1473	methional	6	3

^a^ FD: Flavor dilution factor determined on DB-5ms and DB-wax column. ^b^ SD: Sample dilution factor determined on DB-5ms and DB-wax column.

**Table 4:** Characteristic ion, concentrations, threshold, and odor activity values (OAVs) of the key aroma compounds in doubanjiang after solvent assisted flavor evaporation (SAFE).

No.	Compounds	Characteristic Ion (m/z) ^a^	Concentrations (μg/kg)	Odor Threshold (μg/L in Water) ^b^	OAV ^c^
3	ethyl 3-methylbutanoate	88,57,85	62.29 ± 2.25	0.01	6229
28	limonene	68,93,67	32.36 ± 1.81	200	0.16
64	linalool	71,93,55	33.2 ± 1.42	28	1.18
65	phenylethyl alcohol	91,92,122	129.22 ± 3.61	564.2	0.23
89	β-damascenone	69,121	6.69 ± 0.32	0.002	3345
103	acetic acid	43,45,60	103.2 ± 2.75	22	4.69
106	3-methylbutanoic acid	60,43,87	144.75 ± 4.65	70	2.07
124	3-isobutyl-2-methoxypyrazine	124,151	4.98 ± 0.32	0.002	2490
126	2-methoxyphenol	109,124,81	27.24 ± 1.25	3	9.08
134	3-methylbutanal	44,58	1.13 ± 0.08	1.1	1.03
136	phenylacetaldehyde	91,92,120	6.64 ± 0.43	4	1.66
152	sotolone	83,128	16.02 ± 1.09	0.01	1602
155	methional	48,76,104	32.0 ± 1.07	0.2	160

^a^ The first qualify ion was selected as a quantifying ion in quantitative analysis, which is based on 8-point external standard curves in the SAFE. ^b^ Odor threshold was taken from Volatile Compounds in Food (VCF) database http://www.vcf-online.nl/OFTVCompoundSearch.cfm. Odor thresholds were determined in water. ^c^ OAV value = $\frac{\mathrm{Average}\;\mathrm{concentrations}\;\left(\mu g/\mathrm{kg}\right)\;}{\mathrm{Odor}\;\mathrm{threshold}\;\left(\mu g/L\;\mathrm{in}\;\mathrm{water}\right)}$.

**Table 5:** Effects of key aroma-active compounds on doubanjiang sensory attributes.

No.	Compounds	Effects
3	ethyl 3-methylbutanoate	sweet **, fruity *
28	limonene	fruity *
64	linalool	fruity **, sweet **
65	phenylethyl alcohol	fruity **, sweet **
89	β-damascenone	fruity ***, sweet ***
103	acetic acid	-
106	3-methylbutanoic acid	-
124	3-isobutyl-2-methoxypyrazine	burnt ***, red pepper ***, caramel-like **
126	2-methoxyphenol	caramel-like **, burnt *
134	3-methylbutanal	fruity *
136	phenylacetaldehyde	fruity *, sweet **
152	sotolone	caramel-like ***, fruity **
155	methional	-

“–” means no significant difference. ***, at *p* < 0.001 level. **, at *p* < 0.01 level. *, at *p* < 0.05 level.

## Supplementary Materials

Supplementary File 1
