# Supplementary material for: Characterization and Evaluation of Aroma Quality in Doubanjiang, a Chinese Traditional Fermented Red Pepper Paste, Using Aroma Extract Dilution Analysis and a Sensory Profile
Source: Molecules. 2019 Aug 27;24(17):3107. doi: 10.3390/molecules24173107 (PMC6749255; doi:10.3390/molecules24173107)

molecules

MDPI

Additonal figure

chemical structuresβ-damascenone, 3-isobutyl-2-methoxypyrazine (IBMP), and sotolone

β-damascenone:

- **CAS Registry Number:** 23726-93-4
- **Chemical structure:**

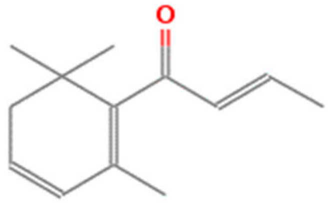

3-isobutyl-2-methoxypyrazine:

- **CAS Registry Number:** 24683-00-9
- **Chemical structure:**

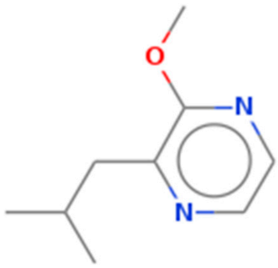

Sotolone:

- **CAS Registry Number:** 28664-35-9
- **Chemical structure:**

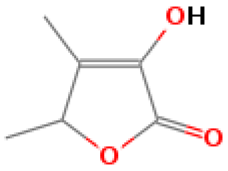

Supplement: Supplementary File 1 [file molecules-24-03107-s001.pdf]
